# Identifying conserved UV exposure genes and mechanisms

**DOI:** 10.1038/s41598-018-26865-9

**Published:** 2018-06-05

**Authors:** Susana I. L. Gomes, Carlos P. Roca, Janeck J. Scott-Fordsmand, Mónica J. B. Amorim

**Affiliations:** 1 Department of Biology & CESAM, University of Aveiro, Aveiro, 3810-193 Portugal; 2 Department of Chemical Engineering, Universitat Rovira i Virgili, Tarragona, 43007 Spain; 3 Department of Bioscience, Aarhus University, Vejlsovej 25, Silkeborg, PO BOX 314, DK-8600 Denmark

## Abstract

Studies have been showing how changes in ultraviolet (UV) affect the terrestrial system, mostly focusing on higher plants and indirect effects, e.g. UV changed food quality/decomposition. Much less attention has been given to direct effect on terrestrial species, although the negative effects have been recognized for some earthworms. Further, the actual mechanisms of UV toxicity to soil invertebrates are even less understood. We here studied the effect of UV on the soil oligochaete *Enchytraeus crypticus*, and attempted to identify the possible mechanisms of toxicity using high-throughput gene expression. Applying a UV dose equivalent to UV during the winter months in northern Europe we observed an 80% decrease in reproduction. For these organisms, approximately 5% of the genes were differentially expressed. Among the observations was an activation of the DNA repair mechanisms, nucleotide excision repair, which correlated with survival of the organisms. An observed repressing of apoptosis seems to have deleterious effects (e.g. because it may lead to the accumulation of aberrant cells) leading to a decline in reproduction. The mechanisms activated by UV were similar to those mechanisms activated in humans, showing conservation across species.

## Introduction

Over the past decades, the increased levels of solar ultraviolet (UV) radiation due to the ozone layer depletion have raised concerns for humans and the environment (e.g.^1,2^).

Considerations on how changes in UV may affect the terrestrial system have been reported, but these studies mostly focus on direct effects on higher plants or, on the indirect effects of UV on other organisms such as earthworms^3,4^. Much less attention has been given to direct effects on terrestrial species^5^. Although terrestrial organisms such as enchytraeids and earthworms generally avoid solar exposure, many species either reside in soil’s upper layers or will surface regularly e.g., after heavy rain falls worms may remain on the soil surface for hours^6^. Surfacing in day-time obviously leads to exposure to sun light and, potentially, also increased UV. Due to their burrowing life style, it is likely that they are not well-adapted to cope with increased UV radiation. Previous studies with *Eisenia fetida* showed a dose dependent decrease in survival, fecundity and total population number, when exposed for 12 hours daily to normal and increased (30% higher) UV radiation^7^. Another study showed that exposing the earthworms *Amynthas gracilis* and *Metaphire posthuma* to UVB (equivalent dose obtained in 1–3 hours on a cloudy day), caused both increased mortality and abnormal behaviour (increased S-shape movements and jumping)^6^. UV radiation also causes histological abnormalities, production of reactive oxygen species (ROS), and lipid peroxidation as shown in the integument of the earthworm species *M*. *posthuma*^8^. Despite the fact that both population and enzyme responses have been observed (see above), nothing is known regarding the UV induced gene-expression changes for these organisms. *In vitro* studies, using human cell lines, indicate where to look for UV effects, i.e. on specific signalling, transcription and RNA metabolism, DNA repair and cell cycle control^9,10^.

We aimed to assess the effects of UV to a soil inhabiting enchytraeid worm (*Enchytraeus crypticus*) at transcriptomic level and link the results to the phenotypic and population level. The organisms were exposed to ecologically relevant UV doses (erythema/effective doses of 204 and 220 J/m^2^) for 15 min daily, which is equivalent 15–30 minutes of sun light in temperate regions^6,11^.

**Figure 1:**
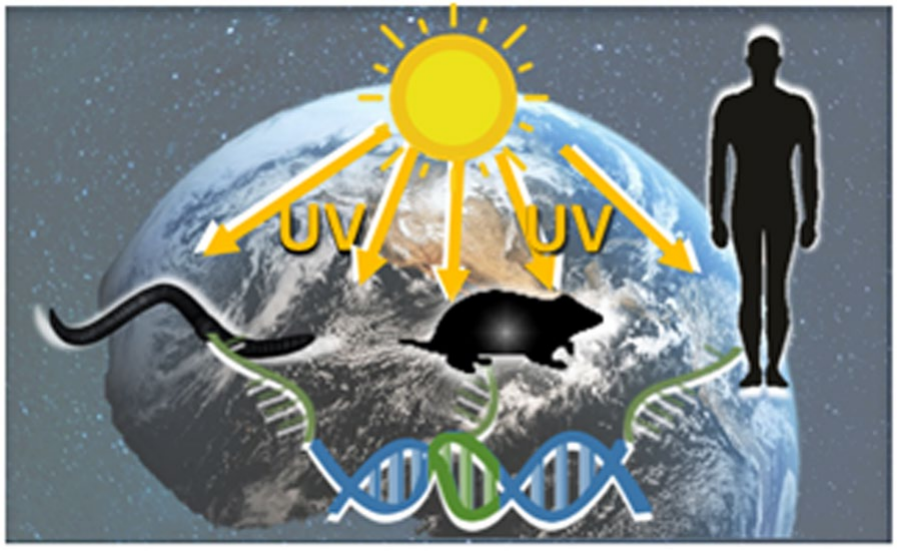
Cross species transcriptomics information transfer: representation of the potential conserved mechanisms to UV exposure and use of alternative species.

The species used, *E*. *crypticus*, is an important representative of the soil fauna, contributing to the improvement of the soil pore structure and to the degradation of organic matter^12^ and they are also model organisms in soil ecotoxicology^13,14^ and transcriptomics^15^. Hence, given the importance of this species the logical consequence is that if UV affects these organisms, it will probably affect the function and structure of the ecosystem (via this or other related species). Further, there is potential for cross-species extrapolation, especially in terms of conserved mechanisms (see schematic Fig. 1).

## Results

### Survival and reproduction

The results for the pre-exposure in reconstituted standard ISO test water (further referred as ISO water) for 5 days showed that survival in controls, at 204 J/m^2^and 220 J/m^2^of UV was 98%, 93% and 92%, respectively.

**Figure 2:**
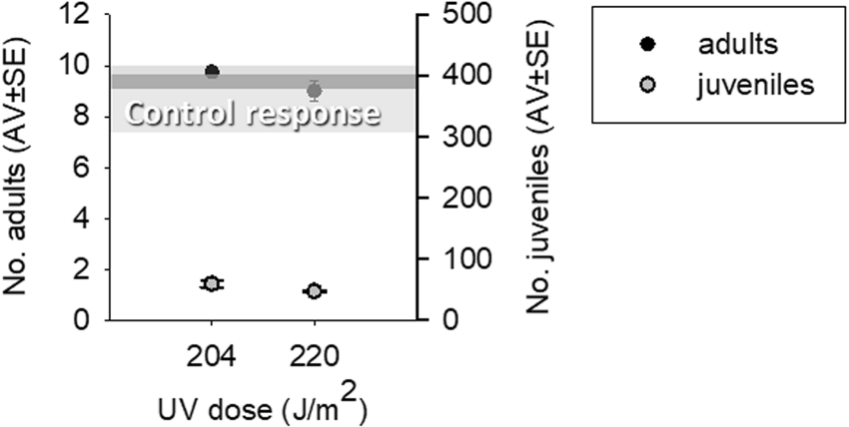
Number of adults and juveniles of *Enchytraeus crypticus* in OECD soil (21 days), after pre-exposure for 5 days, in ISO water to UV radiation at 204 J/m^2^ and 220 J/m^2^ and followed by 21 days in OECD soil. Grey bars represent the control range for survival (medium grey) and juveniles (light grey), with the darker grey showing the overlapping area.

The results of post-exposure in OECD soil are shown in Fig. 2. After 21 days in clean soil, the pre-exposure to UV caused a significant reduction in reproduction by approximately 84% and 87%, for 204 and 220 J/m^2^, respectively, and no significant effects were found on survival.

### Gene expression – microarrays

**Figure 3:**
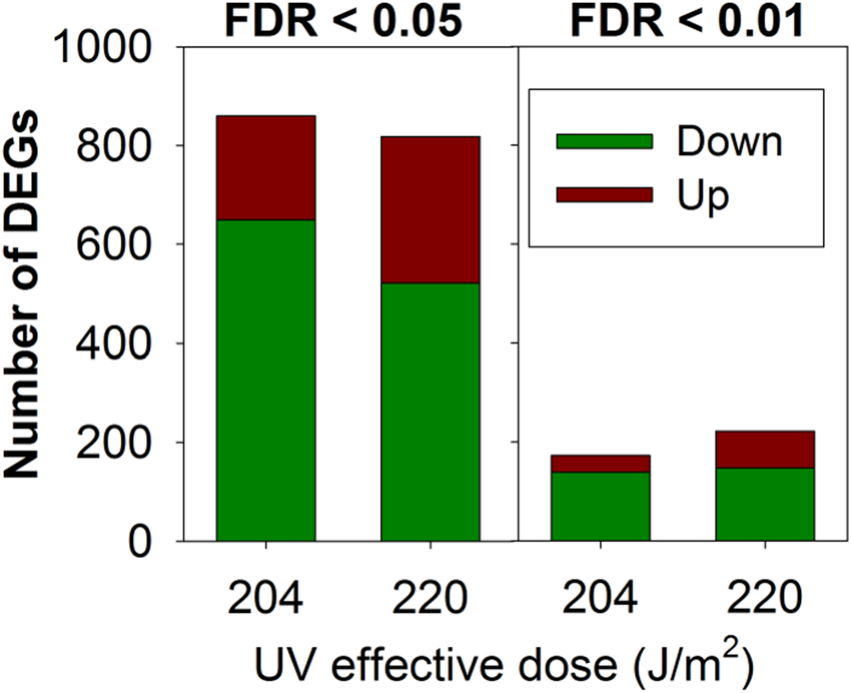
Number of differentially expressed genes (DEGs), controlling the false discovery rate below 5 and 1% (FDR < 0.05 and 0.01) in *Enchytraeus crypticus* affected by exposure to UV radiation at effective doses of 204 and 220 J/m^2^, for 5 days in ISO water. Down/Up: down- or up-regulated.

A total of 1709 (false discovery rate (FDR) < 0.05) transcripts and a total of 249 (FDR < 0.01) transcripts (out of 38516 gene probes with signal detected) were differentially expressed, in at least one test condition (for details see Fig. 3 and Table S1, Supporting Information for the full list of differentially expressed genes (DEGs)).

The number of DEGs decreased as expected around 7 times when considering an adjusted *p* < 0.01, in comparison to adjusted p < 0.05. There were more down- than up-regulated transcripts. Although adjusted p < 0.05 is commonly used, given the somewhat deficient lack of control of the FDR in microarray studies^16^, we opted to be more conservative and selected only the DEGs with FDR < 0.01, in order to reduce the number of false positives among DEGs.

**Figure 4:**
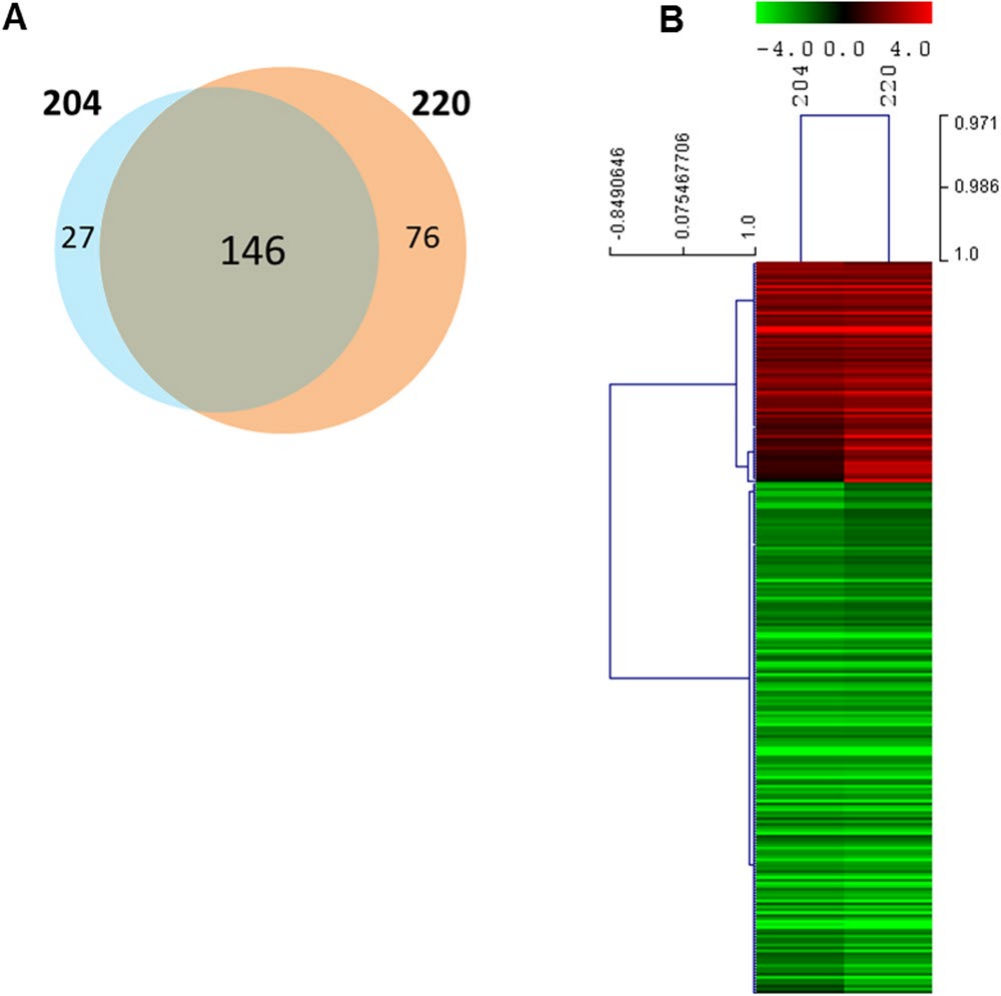
Venn diagram (**A**) and heat map of genes and samples hierarchically clustered using Pearson uncentered and average linkage (**B**) of differentially expressed genes (FDR < 0.01) after exposure to UV radiation at effective doses of 204 and 220 J/m^2^, for 5 days in ISO water.

The Venn diagram (Fig. 4A) depicts the number of DEGs that are shared and exclusive among UV doses. Results showed a high number of overlapping genes between the two UV doses (146), as also reflected in the heat map results (Fig. 4B), where a closely related transcriptional response between the two UV doses (97% similarity) is shown.

Enrichment analysis on Gene Ontology (GO) terms was performed to identify biological processes significantly affected by each UV dose. For that, the gene lists containing the transcripts significantly affected by each UV dose were analysed, using as background the list of genes with signals detected in microarrays (38516 gene probes). The most specific GO terms (of the category Biological Processes), among those significantly affected (Fisher’s Exact Test *p* < 0.05) in each test condition are listed in Table 1.

**Table 1:** Significantly enriched (Fisher’s Exact Test *p < *0.05) Gene Ontology terms at the two UV effective doses: 204 and 220 J/m^2^. GO: Gene Ontology; #Sig: number of significant sequences; #Annot: number of annotated sequences.

GO-ID	Biological process	p-value	#Sig	#Annot
**204 J/m2**				
GO:0031100	animal organ regeneration	0.002	2	18
GO:0017187	peptidyl-glutamic acid carboxylation	0.003	1	0
GO:0010971	positive regulation of G2/M transition of mitotic cell cycle	0.003	1	0
GO:0046602	regulation of mitotic centrosome separation	0.003	1	0
GO:0007144	female meiosis I	0.003	1	0
GO:0032571	response to vitamin K	0.003	1	0
GO:0060212	negative regulation of nuclear-transcribed mRNA poly(A) tail shortening	0.003	1	0
GO:0071373	cellular response to luteinizing hormone stimulus	0.007	1	1
GO:0048642	negative regulation of skeletal muscle tissue development	0.007	1	1
GO:0007307	eggshell chorion gene amplification	0.007	1	1
GO:0001701	in utero embryonic development	0.007	3	114
GO:0043486	histone exchange	0.010	1	2
GO:0033574	response to testosterone	0.017	1	4
GO:0006323	DNA packaging	0.017	2	57
GO:0019919	peptidyl-arginine methylation, to asymmetrical-dimethyl arginine	0.023	1	6
GO:0006261	DNA-dependent DNA replication	0.025	2	70
GO:0048255	mRNA stabilization	0.027	1	7
GO:0008584	male gonad development	0.030	1	8
GO:0040016	embryonic cleavage	0.030	1	8
GO:0040020	regulation of meiotic nuclear division	0.036	1	10
GO:0045948	positive regulation of translational initiation	0.040	1	11
GO:0006378	mRNA polyadenylation	0.043	1	12
GO:0045740	positive regulation of DNA replication	0.043	1	12
GO:0006265	DNA topological change	0.049	1	14
GO:0009303	rRNA transcription	0.049	1	14
GO:0046622	positive regulation of organ growth	0.049	1	14
**220 J/m2**				
GO:0031100	animal organ regeneration	0.003	2	18
GO:0010971	positive regulation of G2/M transition of mitotic cell cycle	0.004	1	0
GO:0060212	negative regulation of nuclear-transcribed mRNA poly(A) tail shortening	0.004	1	0
GO:0071373	cellular response to luteinizing hormone stimulus	0.008	1	1
GO:0048642	negative regulation of skeletal muscle tissue development	0.008	1	1
GO:0007307	eggshell chorion gene amplification	0.008	1	1
GO:0006754	ATP biosynthetic process	0.018	3	128
GO:0048639	positive regulation of developmental growth	0.020	2	50
GO:0033574	response to testosterone	0.021	1	4
GO:0030321	transepithelial chloride transport	0.025	1	5
GO:0019919	peptidyl-arginine methylation, to asymmetrical-dimethyl arginine	0.029	1	6
GO:0048255	mRNA stabilization	0.033	1	7
GO:0008584	male gonad development	0.037	1	8
GO:0040016	embryonic cleavage	0.037	1	8
GO:0006972	hyperosmotic response	0.041	1	9
GO:0006260	DNA replication	0.045	3	185
GO:0045948	positive regulation of translational initiation	0.049	1	11

Results showed that 204 J/m^2^ of UV affected more biological processes than 220 J/m^2^ (26 and 17, respectively), even though the number of transcripts affected by 204 J/m^2^ was slightly smaller. Several processes were affected across conditions, i.e., animal organ regeneration, embryonic cleavage, male gonad development, response to testosterone, mRNA stabilization, positive regulation of translational initiation, etc. Specifically affected by 204 J/m^2^ of UV are processes such as: DNA packing and DNA topological change, female meiosis I, histone exchange, in utero embryonic development and response to vitamin K. Specifically affected by 220 J/m^2^ of UV are: ATP biosynthetic process, hyperosmotic response and transepithelial chloride transport.

Additionally, the list of DEGs (FDR < 0.01), annotated and varying in expression more than 4 fold (additional cut-off) in at least one test condition (i.e., |M| > 2) is shown in Table S2 (Supporting Information), including the information of the KEGG (Kyoto Encyclopedia of Genes and Genomes) pathways in which each gene is involved. The following pathways were commonly affected by both UV doses: Arachidonic acid metabolism, Nucleotide excision repair, Neuroactive ligand-receptor interaction by up-regulated DEGs; and RNA transport, mRNA surveillance pathway, RNA degradation, FoxO signalling pathway and Glucagon signalling pathway by down-regulated DEGs. The pathways cGMP-PKG signalling, cAMP signalling, Adrenergic signalling in cardiomyocytes, Insulin secretion, Salivary secretion and Pancreatic secretion were linked to the transcript plasma membrane calcium-transporting ATPase 3, significantly up-regulated by 220 J/m^2^ of UV, and to the transcript sodium potassium-dependent ATPase beta-2 subunit, significantly down-regulated by 204 J/m^2^. This is discussed below besides the EA output.

**Figure 5:**
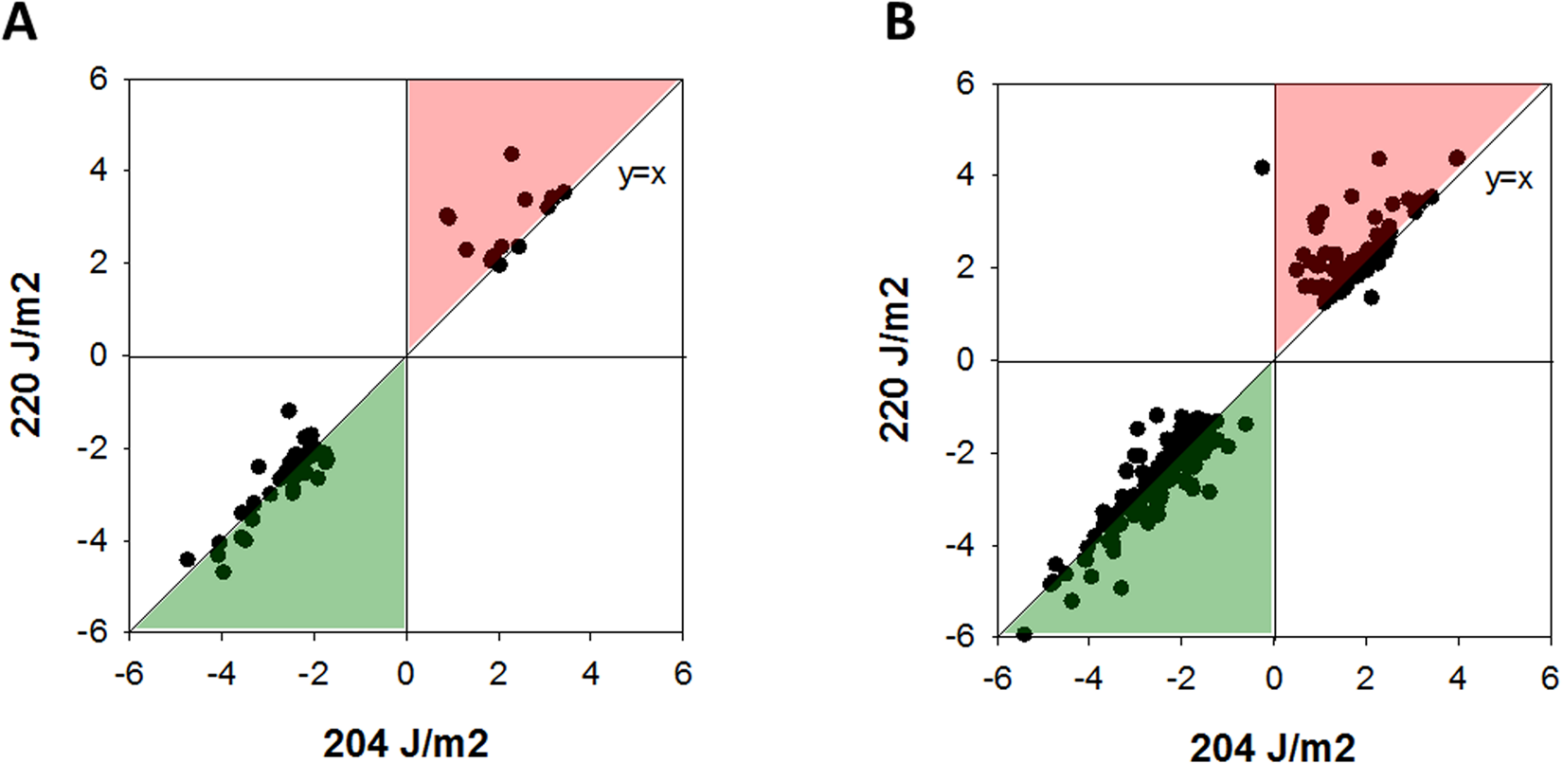
Plot of gene expression values for 204 and 220 J/m^2^ of UV for (**A**) the DEGs presented in S2 Table and (**B**) all the DEGs (FDR < 0.01). The points in the red area (above the line x = y) are transcripts with higher expression with increased dose, the points in the green area (below the line x = y) are transcripts with lower expression with increased dose.

Expression values of DEGs are plotted in Fig. 5 to show the relative increase in the absolute values with the increase in the exposure dose. This dose-response pattern is more pronounced for the up-regulated genes (Fig. 5, red area). The dose-response associated genes are marked grey in Table S2.

## Discussion

In this study two key observations were made, (i) UV exposure at what would be considered short-term and low-doses can cause serious population effects on soil-inhabiting worms, and (ii) the related gene expression changes are similar to responses shown for humans. Whereas (i) indicate that even a little increase in UV light, e.g. due to global changes, may have significant consequences for the soil species, the (ii) observation indicates that the mechanisms behind UV effects are likely highly conserved and can be used to study similar impacts on other organisms.

Regarding the population effects: The tested UV radiation caused an 80% decrease in enchytraeid reproduction, whereas survival was not affected (less than 10%). This decline occurred following an exposure of 15 min daily 204/220 J/m^2^ pulse (for 5 days). This UV dose falls within a realistically exposure comparable to what is measured during the winter months in the UK^11^, and is similar to doses delivered in less than 30 min on a cloudy day in temperate regions^6^. Hence, our results show that a short daily “realistic” exposure over 5 days is enough to cause pronounced damage at later life stages, as measured by the endpoint reproduction. This initial UV damage is not reversed during the 21 days with no UV exposure. These results are in agreement with those observed in previous UV studies^17^. Effects on mortality start to occur around 200–220 J/m^2^ (ca. 10%), yet at that dose the reproduction capacity is already seriously impacted. The reproductive effect observed on worms combined with knowledge on increased levels of UV radiation globally^18,19^, indicate that the geographical distribution of enchytraeid species can be affected. This impact will vary in nature, depending on e.g. time of the year, canopy coverage, latitude, weather or natural variability in the ozone layer, but it will still prevail. This organismal effect may either have caused a secondary effect on reproduction or, given the severity of the reproductive effects, the UV had a direct effect on the reproductive organs/cells. At 312 nm (intensity peak of the UV lamp used), UV radiation penetrates around 50–60 μm in human skin (skin phototype I, pale white skin)^20^. Knowing that *E*. *crypticus* epidermis is around 15 μm^21^, the UV radiation can reach their internal organs. Additionally, enchytraeids are transparent which would increase the UV penetration. Although not an easy task, this may be confirmed by the egg/sperm-analysis (but *E*. *crypticus* can reproduce sexually and by fragmentation^22^).

Although earthworms may avoid direct sun light, they will experience UV exposure regularly, for instance after heavy rainfalls when they are often found on the soil surface^6^. Terrestrial enchytraeids [enchytraeid species can be found in several habitats, including marine ones^23^] live in the top layers of soils; in forested areas, some enchytraeid species live in the upper 2 cm of soil during all year^24^. In these cases, and due to their burrowing activity, enchytraeids contribute to the soil pore structure, for instance leading to the rehabilitation of sealed soil surfaces by rainfalls^25^. Unlike other soil oligochaetes, most enchytraeid species are transparent-whitish, lacking a keratinised layer protecting against UV radiation and hence are in principle more susceptible than earthworms which often have skin pigments, and even more than in human skin. This area is not very explored but the findings in our study show that UV is an important factor to consider, especially under a climate change scenario that can affect UV radiation reaching the Earth. This is not only directly related with ozone but also related with other factors such as changes in aerosols, clouds, or surface reflectance^19^. Further, as mentioned, the increase in UV radiation may affect the species geographical distribution (i.e., via the impediment of their reproductive ability).

Since the experiments are performed with a population that has been cultured in the laboratory for years in none- or low-UV conditions, these organisms may have lost some of their previous UV protection mechanisms. However this problem is the same for both human health and environmental studies where laboratory cultures are always used, and could be an issue in virtually all possible effects studies. This means that the results may be over-estimating effects compared to natural populations but the mechanisms should be the same.

Regarding the mechanisms: The overall gene expression profiles (pathways or GO terms) were similar between the two UV doses, with 5% of the genes being differentially expressed (DEGs). These DEGs are probably potential gene-pattern markers for UV exposure that can be used as endpoints when assessing the risk of UV radiation. The KEGG pathways for DEGs common to 204 and 220 J/m^2^ showed that the arachidonic acid (AA) metabolism was affected by both UV doses, given the up-regulation of prostaglandin d2 hematopoietic-like. This corresponds to human studies, where increased concentrations of AA and prostaglandins D2 (and also E2 and F2α) were also observed in human skin exudates after exposure to UV-B radiation^26^. These results indicate a potentially conserved mechanism of response to UV. More recently, it has been shown that following UV exposure, AA produces AA-peroxides, which induce apoptosis in Neuron-2A cells at higher levels than non-irradiated AA^27^. Additionally, UV irradiated AA caused mitochondria malfunctions and induced a rise in Ca^2+^ intracellular levels in Neuron-2A, which could be implicated in the increased cellular apoptosis^27^. The calcium signalling pathway (and the calcium dependent: cGMP-PKG and cAMP signalling pathways) was also affected in 220 J/m^2^, apparently towards the export of Ca^2+^ out of the cells (by the up-regulation of plasma membrane calcium-transporting ATPase 3), indicating an anti-apoptotic response.

Also affected by both UV doses was the nucleotide excision repair, which in humans is a recognized mechanism of defence against the effects of UV-B radiation (e.g.,^28^). Maeda *et al*.^28^ showed that nucleotide excision repair (NER) genes were up-regulated in human keratinocytes after exposure to 100 J/m^2^ of UV, being responsible for the complete DNA repair within 24 h and prevention of apoptosis. On the contrary, they observed that at doses of 300 and 600 J/m^2^ the NER genes were down-regulated and possibly related with the prevalence of the apoptotic pathway^28^. Our current results showed that, in enchytraeids, UV doses of around 200 J/m^2^ activate mechanisms of DNA repair and not apoptosis.

RNA degradation, RNA transport and mRNA surveillance pathways were associated with the transcript poly-A binding cytoplasmic 1 b (PABPC1) down-regulated by both UV doses. PABPC1 is required for mRNA poly A shortening, a crucial step in mRNA degradation and which was found inhibited by UV-B radiation^29^. The inhibition of mRNA degradation, i.e., RNA stabilization, described at 600 J/m^2^ of UV radiation (and considered low doses by the authors) can lead to the increased expression of proteins derived from normally unstable mRNAs (e.g., inflammatory cytokines, growth factors and proto-oncogenes) greatly conditioning the gene expression profile in response to UV^29^.

The down-regulation of protein arginine methyltransferase 1 (PRMT1) by both UV doses can be implicated in FoxO and Glucagon signalling pathways. The effects of PRMT1 inhibition on glugacon signalling would lead to a reduction in gluconeogenesis suggesting alterations in sugar metabolism. On the other hand, the possible implications of PRMT1 down-regulation on FoxO signalling are a decrease of FoxO1 methylation which would increase cell survival (by decreasing apoptotic stimuli)^30^.

Uniquely up-regulated by 220 J/m^2^ is the 3-phosphoinositide dependent protein kinase-1 (PDK1) which can be involved in several pathways where it participates for instance in the transduction of signals from insulin to control cell proliferation and survival, or in glucose and amino acids uptake and storage and in Ca^2+^ entry regulation in immune cells, etc. It has been shown that UV-B can activate PDK1, which plays a central role in the activation of protein kinase B (or Akt), which in turn allows the survival of skin keratinocytes exposed to UV^31^. This is an anti-apoptotic mechanism that leads to the accumulation of aberrant cells that can evolve to cancerous cells^31^. PDK1 is also involved in mTOR signalling pathway, as well as the transcript ras-related gtp-binding protein a (RRAGA), down-regulated by 220 J/m^2^. mTOR plays roles in cell growth and cell cycle, control of the cytoskeleton and nutrient transport, protein and RNA stability, and transcription and translation. This is also known to be impaired in response to stressful stimuli including DNA damage^32^. In our study, both the down-regulation of RRAGA and the up-regulation of PDK1 (which phosphorylates Akt, acting as an mTOR inhibitor) indicate the inhibition of the mTOR signalling pathway.

The CCAAT enhancer-binding protein beta (C/EBPβ), significantly up-regulated by 220 J/m^2^, can be involved in TNF signalling pathway, which is a pathway that can induce a wide range of intracellular signal pathways, including apoptosis and cell survival as well as inflammation and immunity. The levels of C/EBPβ protein were induced in mice keratinocytes after exposure to UV-B in a study where UV-B was applied as a DNA damaging agent, and C/EBPβ responds to DNA damage to promote cell survival by repressing p53 levels^33^.

Unique to 204 J/m^2^ was the down-regulation of the Na/K dependent ATPase beta-2 subunit (ATP1B2), which can be involved in several pathways, including the cGMP-PKG signalling pathway, cAMP signalling pathway, cardiac muscle contraction, insulin secretion, etc. ATP1B2 is part of an integral membrane protein responsible for establishing and maintaining the electrochemical gradients of Na^+^ and K^+^ across the plasma membrane. It is not clear how its down-regulation is related to further effects in enchytraeids, however UV-B radiation induced changes in membrane potential *in vitro*^34^. A Na/K-dependent ATPase (ATP1A1) was also found down-regulated in UV exposed HaCaT cells^10^.

The results from the Enrichment Analysis of GO terms are in good agreement with the KEGG pathways in which the DEGs can be involved. For instance, the GO terms mRNA stabilization and negative regulation of nuclear-transcribed mRNA poly (A) tail shortening reflect the inhibition of RNA degradation by the down-regulation of PABPC1. In addition to the pathways previously discussed, GO terms associated with the reproductive system and embryo development, i.e., female meiosis I and regulation of meiotic nuclear division (unique to 204 J/m^2^) and male gonad development and embryonic cleavage (common to 204 and 220 J/m^2^) indicate that UV can have direct effects of reproductive cells/tissues which could have contributed to the observed reproductive effects (more than 80% reduction in reproduction).

Our results indicate that, at the transcript level, UV exposure is activating DNA repair mechanisms while inhibiting RNA degradation. The general mechanisms seem for a large extent to be similar to those in humans, and hence must be conserved mechanisms. In enchytraeids exposed to UV (204 and 220 J/m^2^), cell survival is being promoted in detriment of apoptosis, which can lead to the accumulation of aberrant cells and cause further damage to the organisms. In this case we measured a high effect in reproduction. Given that effects were present in the post-exposure part there must be a direct effect of UV on the organisms.

The gene response patterns induced by UV in the worms seem to be similar to those observed for humans, indicating that these responses are widely conserved across species. If scepticism remains about the relevancy of exposure of soil organisms to UV, the present study provides indications of the basic mechanisms to UV that should be studied in other soil organisms.

## Conclusions

Exposure to UV (doses equivalent to the winter months in northern Europe) caused 80% decrease in reproduction, i.e. they had a very high impact. An increase in UV can affect the natural populations’ dynamics, risking enchytraeids geographical distribution. High-throughput gene expression showed that the mechanisms of response to UV must be well conserved between species, as shown by the similarity to other species including humans. This is interesting to observe for species that naturally avoid sun (UV) exposure. UV triggered DNA repair mechanisms and promoted cell survival (repressing apoptosis). While the first promotes survival of the organisms, the latter has deleterious effects and may lead to the accumulation of aberrant cells resulting in negative apical effects like the one measured in terms of reproduction.

## Materials and Methods

### Test species

The standard test organism *Enchytraeus crypticus*^21^ was used. Individuals were maintained in laboratory cultures for many years, cultured in Petri dishes containing agar medium, consisting of a sterilized mixture of four different salt solutions (CaCl_2_·2H_2_O; MgSO_4_; KCl; NaHCO_3_) and a Bacti-Agar medium (Oxoid, Agar No. 1). Cultures were kept under controlled conditions, at 19 °C and photoperiod cycle of 16:8 hours light:dark (light provided by fluorescent lamps, see Fig. S1 for the spectrum, SI), and they were fed on ground autoclaved oats twice a week.

### Test media

Reconstituted standard ISO test water^35^ was used for the exposure to UV (and control), containing: 2 mM of CaCl_2_.2H_2_O, 0.5 mM of MgSO_4_.7H_2_O, 0.77 mM of NaHCO_3_ and 0.077 mM of KCl in ultra-pure water. The exposure procedure followed the indications in water test^36^ as a surrogate to ensure exposure to the UV light although less realistic.

Organisation for Economic Co-operation and Development (OECD) artificial soil was used for the post-exposure. The soil was prepared according to the guideline^13^ containing: 75% of sea sand (VWR, technical wash with sulphuric acid), 20% of Kaolin clay (Sigma Aldrich), 5% of sphagnum peat (sieved to ≤ 2 mm) and CaCO_3_ (Merck) for pH adjustment to 6 (±0.5). All the soil constituents were mixed thoroughly and allowed to stabilize (1 week) prior to use.

## Experimental Details

### UV exposure and testing

Organisms were exposed to UV radiation, 15 min on a daily basis (for 5 days), corresponding to ecologically relevant low UV doses (204 and 220 J/m^2^). For instance, in the centre of UK, erythema doses in January are around 125 J/m^2^ and around 2400 J/m^2^ in July^11^; in equatorial regions doses range from 5000 to 7000 J/m^2^, recorded in June of 2007^37^.

Hence, for the two exposures, the UV irradiances (280–400 nm) were equivalent to 1670 ± 51 and 1800 ± 43 mW/m^2^. UV effective doses were calculated as: UVD*eff*(J/m^2^) = [I*eff* (mW/m^2^) × time (sec.)]/1000, the effective UV irradiance (I*eff*) being corrected by the weighting factors of the CIE reference action spectrum for erythema in human skin^38^. For that, the intensity across the radiation spectrum (280–400 nm) was measured, at intervals of 1 nm, with a spectroradiometer connected to a monochromator and analysed with BenWin + software (Bentham Instruments, Reading, UK). Each wavelength was multiplied by the corresponding CIE weighting factor^38^, and summed up to obtain the total UV irradiance.

Ultraviolet radiation was provided by a UV lamp (Spectroline XX15F/B, Spectronics Corporation, NY, USA, peak emission at 312 nm) and a cellulose acetate sheet was coupled to the lamp to cut-off UVC range wavelengths (because this is radiation that does not reach the earth’s surface). The spectrum of the lamp is provided in Fig. S2 (SI). The two doses were obtained by varying the distance of the UV lamp to the test vessels, i.e., the lamp was placed at 43 and 46 cm above the test vessels to obtain 220 and 204 J/m^2^ of UV, respectively.

The test conditions were as described in Gomes *et al*.^17^. In summary, the exposure was performed in 24 well plates, each well corresponds to a replicate and contained 1 ml of ISO water and five adult organisms with *clitellum*. The test duration was five days, at 20 ± 1 °C. Survival was assessed every 24 hours, and organisms were considered dead when not responding to any mechanical stimulus. Ten replicates for each test condition were performed (50 organisms per treatment), including controls without UV irradiation (fluorescent light, the standard conditions). The test plates were kept at a photoperiod of 16:8 h light:dark, except for the 15 min of daily UV irradiation performed in the middle of the “light” period. Note that these are ecologically relevant UV doses: erythema/effective doses of 204 and 220 J/m^2^ for 15 min daily is equivalent to 15–30 minutes of sun light in temperate regions^6,11^.

#### Post-exposure in OECD soil

After exposure in ISO water to UV irradiation (5 days), organisms were transferred to OECD soil. The procedure followed the Enchytraeid Reproduction Test (ERT) guideline^13^ (i.e., 21 days post-exposure). The surviving organisms for each test condition were pooled in groups of 10 and introduced in test vessels with moist soil (50% of maximum Water Holding Capacity - mWHC) and 25 mg of food (ground oats). Four replicates per pre-exposure condition were performed, each having 10 organisms. The vessels were covered with a lid containing small holes and the test ran for 21 days, at 20 ± 1 °C and 16:8 hours photoperiod. Weekly, 12.5 mg of food was supplied, and soil moisture was adjusted by replenishing weight loss. At the end, adult enchytraeids were searched in the soil under a stereo microscope and counted. Juvenile enchytraeids were immobilized with ethanol and coloured with Bengal red (1% solution in ethanol). Each replicate was sieved to eliminate the kaolin clay and then juveniles were counted under a stereo microscope.

### Gene expression: microarrays

The organisms were exposed to the same conditions previously described above (i.e., control: CT/no UV radiation, and UV effective doses of 204 and 220 J/m^2^), during 5 days. Thirty-two replicates per test condition (each corresponding to a well plate containing 5 organisms) were performed, to obtain 4 samples for RNA extraction, with 40 organisms each. At the end of the test, organisms were carefully removed from the ISO water, frozen in liquid nitrogen and stored at −80 °C until further analysis.

#### RNA extraction, labelling and hybridizations

Total RNA was extracted (3 samples out of the 4 were used) using SV Total RNA Isolation System (Promega). The quantity and purity of the isolated RNA were measured spectrophotometrically with a nanodrop (NanoDrop ND-1000 Spectrophotometer), and its quality was checked on a denaturing formaldehyde agarose gel electrophoresis. A single-colour design was used. In brief, 500 ng of total RNA was amplified and labelled with Agilent Low Input Quick Amp Labelling Kit (Agilent Technologies, Palo Alto, CA, USA). Positive controls were added with the Agilent one-colour RNA Spike-In Kit (Agilent Technologies, Palo Alto, CA, USA). Purification of the amplified and labelled cRNA was performed with the RNeasy columns (Qiagen, Valencia, CA, USA).

The cRNA samples were hybridized on a Custom Gene Expression Agilent Microarray (4 × 44k format) for this species^39^. Hybridizations were performed using the Agilent Gene Expression Hybridization Kit (Agilent Technologies, Palo Alto, CA, USA), and each biological replicate was individually hybridized on one array. The arrays were hybridized at 65 °C with a rotation of 10 rpm, during 17 h. After that, the microarrays were washed using Agilent Gene Expression Wash Buffer Kit (Agilent Technologies, Palo Alto, CA, USA) and scanned with the Agilent DNA microarray scanner G2505B (Agilent Technologies).

### Acquisition and microarray data analysis

Fluorescence intensity data was obtained with Agilent Feature Extraction Software v. 10.7.3.1 (Agilent Technologies). Quality control was done by inspecting the reports on the Agilent Spike-in control probes. Background correction was provided by Agilent Feature Extraction software v. 10.7.3.1, using recommended protocol GE1 107 Sep09. Only probes with good signal quality (flag IsPosAndSignif = True) were used. Analyses were performed with R^40^ v. 3.3.1 and Bioconductor^41^ v. 3.3 package limma^42^ v. 3.28.20. Data was normalized with SVCD normalization^16^. Differential expression between control and treated samples was assessed with limma methodology. The Benjamini–Hochberg’s (BH) method^43^ was used for multiple testing correction between genes, controlling the false discovery rate (FDR) at two levels: expected FRD < 0.05 and FDR < 0.01, independently for each comparison of treatment versus control). The Minimum Information About a Microarray Experiment (MIAME) compliant data from this experiment was submitted to the Gene Expression Omnibus (GEO) at the National Center for Biotechnology Information (NCBI) (platform: GPL20310; series:GSE69794).

Annotation of microarray gene probes to the Kyoto Encyclopedia of Genes and Genomes (KEGG)^44^ was performed with the KEGG Automatic Annotation Sever (KAAS) v. 2.1^45^, using the representative set for eukaryotic species. Cluster analysis on differentially expressed genes was performed using MultiExperiment Viewer (MeV, TIGR). The differentially expressed genes for each treatment were analysed separately for enrichment analysis of GO (Gene Ontology) terms^46^ using the Blast2GO software.

### Enchytraeid Reproduction Test data analysis

One Way Analysis of Variance (ANOVA), with Dunnett’s method for multiple comparisons at a significance level of 95% (SigmaPlot 11.0), was used to assess differences between the control and each treatment.

## Electronic supplementary material

Emission spectra

Lists of differentially expressed genes
