# Supplementary material for: Identifying conserved UV exposure genes and mechanisms
Source: Sci Rep. 2018 Jun 5;8:8605. doi: 10.1038/s41598-018-26865-9 (PMC5988748; doi:10.1038/s41598-018-26865-9)
Supplement: Supplementary file 1 — Emission spectra [file 41598_2018_26865_MOESM1_ESM.doc]

**Supplementary Information**

**Identifying conserved UV exposure genes and mechanisms**

Susana I. L. Gomes, Carlos P. Roca, Janeck J. Scott-Fordsmand

and Mónica J. B. Amorim


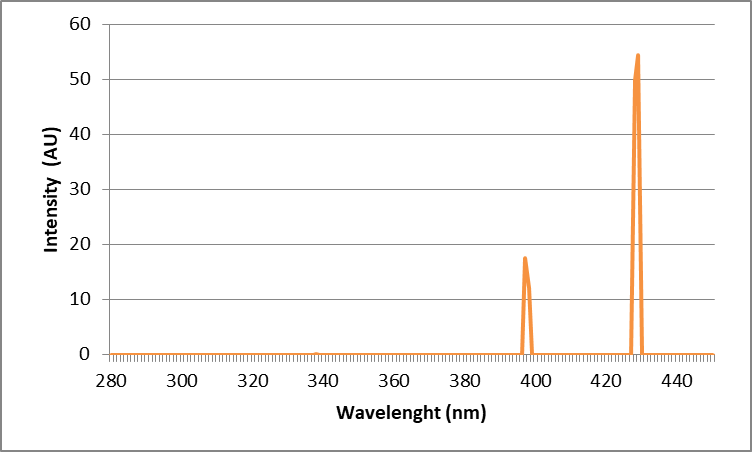


**Fig. S1.** Emission spectra of the fluorescent lamp used for the No-UV exposure. AU: arbitrary units.


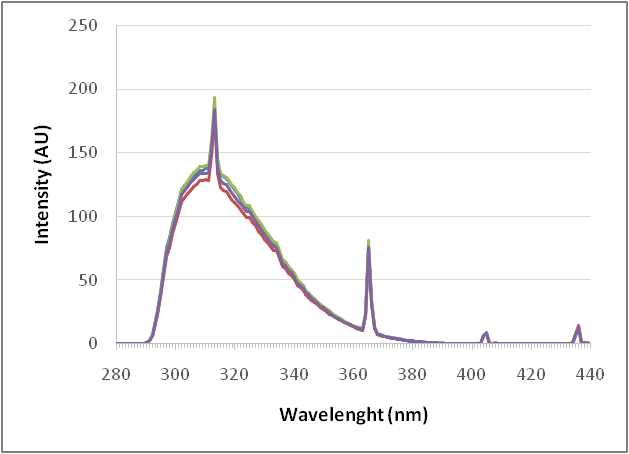


**Fig. S2.** Emission spectra of the lamp used to provide UV radiation (Spectroline XX15F/B lamp, peak at 312 nm). The different colours correspond to the measurements performed in the different days. AU: arbitrary units.
